# Applications of bamboo fiber and bamboo stem ash with styrene butadiene rubber in cement mortar for sustainable structural application

**DOI:** 10.1038/s41598-025-09337-9

**Published:** 2025-08-09

**Authors:** Prem Kumar Vagestan, Manikandan Periyasamy, V. Vasugi

**Affiliations:** 1 Faculty of Engineering and Technology, Villa College QI Campus, Male, Maldives; 2 School of Civil Engineering, Vellore Institute of Technology, Chennai, 600127 Tamil Nadu India

**Keywords:** Bamboo fiber, Bamboo stem Ash, Taguchi optimization, Compressive strength, Microstructure, Civil engineering, Engineering, Materials science

## Abstract

In several developing countries, rapid population growth has led to a shortage of adequate housing. This issue is further intensified by the overuse of traditional construction materials and the environmental concerns linked to their production, particularly the high levels of carbon dioxide emissions. Consequently, the search for environmentally sustainable alternatives has gained momentum. Bamboo, a traditional building resource, has re-emerged as a promising material due to its potential application in construction. This study explored the replacement of Ordinary Portland Cement (OPC) in mortar with bamboo stem ash and alkali-treated bamboo fibers. Various mix ratios were developed and analyzed using the Taguchi method, and the results were validated through analysis of variance (ANOVA), microstructural observations, and energy efficiency evaluation. The optimized mixes demonstrated notable improvements compared to conventional mortar, with an increase in compressive strength of up to 98.42%, flexural strength gains of up to 24%, a 21.7% decrease in dry density, and water absorption limited to 1.52%. Important characteristics of mortar mixes reinforced with bamboo fibers, including as compressive strength, flexural strength, water absorption, and dry density, were efficiently evaluated using the Taguchi technique and is recommend for sustainable bamboo reinforced wall panels.

## Introduction

Bamboo, a fast-growing plant belonging to the Poaceae family of monocotyledonous flowering species, typically matures within two to three years and has been widely used in various construction applications^1–3^. The rapid increase in global population has led to the steady depletion of natural resources, especially raw materials crucial for the construction sector, resulting in a continuous rise in their costs^4,5^. However, the process of manufacturing one metric ton of ordinary Portland cement (OPC) is associated with the release of approximately 0.94 metric tonnes of carbon dioxide emissions into the surrounding environment^6^. Considering the environmental issues linked to cement production, there is growing interest in identifying sustainable options, such as using natural materials like ashes, as potential substitutes for cement to help lower its carbon emissions^7,8^. Recent studies have investigated the application of a wide range of natural by-products^9^, such as fly ash^10^, rice husk ash, silica fume^11^, metakaolin^12^, ground granulated blast furnace slag^13^, marble powder^14^, and egg shell powder^15^, as feasible substitute materials for traditional ordinary Portland cement. Adopting alternative construction materials expands available options and contributes to the broader goal of enhancing sustainability in the construction sector. One such example is the incorporation of bamboo derivatives like stem ash, leaf ash, and fibers, a naturally occurring pozzolanic material, in the production of concrete structural elements.

The use of bamboo and its derivatives in construction has been widely explored in academic research^16^. Bamboo fiber (BF), extracted from the culm through manual peeling, is often added to cementitious materials to improve mechanical performance^17^. Bamboo stem ash (BSA), produced by incinerating bamboo stems and leaves, exhibits strong pozzolanic activity, making it a viable partial cement substitute^18^. The incorporation of BF and BSA into cement mortar and concrete formulations has been demonstrated to to enhance compressive and flexural strength. Additionally, their incorporation contributes to reduced density and water absorption, resulting in lighter composite material with lower water retention^19^. BSA containing high levels of silica and alumina, reacts with calcium hydroxide (CH) to produce calcium silicate hydrate (CSH and calcium aluminate hydrate (CAH), thereby improving the microstructure and durability of cement-based materials. Although it may slow initial hydration, BSA lowers water absorption and enhances long-term performance. Optimal results are typically seen at 5–10% replacement, with some studies supporting effectiveness up to 20%. Further investigation is required to understand its long-term effects on hydration behavior and durability.

Recent investigations have demonstrated that partial replacement of cement with BSA can significantly improve mortar performance. For instance, substituting up to 10% of cement with BSA resulted in a 28% increase in compressive strength at 7 days and an 81% increase at 28 days^20^. However, the same study also reported that when the replacement level was increased beyond 10%, specifically in the range of 10–15%, a reduction in compressive strength was observed. This decline is likely due to the dilution effect and excess unreacted silica, which can interfere with proper hydration and microstructural development. These findings highlight that while 10% BSA serves as an optimum dosage for strength enhancement, higher percentages may require additional modifications. Notably, the use of superplasticizers has been shown to counterbalance this decline by improving particle dispersion and matrix compaction^21^. The progress of bamboo fiber composites from 4 to 16% enhances both the fracture toughness and impact resistance of the composite to a certain degree, as outlined in previous research^22^. Moreover, when bamboo fiber is treated with silane, it demonstrates enhanced flexural and tensile capabilities; however, the incorporation of silane alters the strength properties of concrete^23^. It is noteworthy that cellulose-rich bamboo fibers have been found to reduce the thickness of swelling^24^. Additionally, the age of bamboo is directly related to the quality of its fibers^23^. Specifically, a comparison between one-year-old bamboo fiber and bamboo fiber aged between three and five years reveals the superior characteristics of the latter^25^.

Studies have recently been carried out independently, focusing on the utilization of bamboo fiber as an additional component in mortar and concrete mixtures. It is important to highlight the limited amount of research dedicated to exploring the substitution of bamboo stem ash with cement in varying proportions^18^. Nonetheless, there is a distinct lack of experimental initiatives, focused on evaluating the effectiveness of incorporating bamboo stem ash as an additional binding agent in bamboo-reinforced mortar mixtures.

The compressive strength of mortars gets affected with the increase in the percentage of natural fibers due to fiber-matrix interface, fiber dispersion, fiber length and orientation, fiber volume fraction, types of fiber, properties of fiber and the curing conditions^26^. Alkaline treatment of bamboo fibers with NaOH induces several molecular level changes that enhance their compatibility with cementitious matrices. The process removes surface waxes and oils, exposing additional hydroxyl (-OH) groups, which increases the chemical reactivity of the fibers. NaOH also degrades hemicellulose into simpler sugars and partially disrupts the lignin structure, improving matrix accessibility^27^. Moreover, it alters cellulose crystallinity, making the fiber more amorphous and reactive. These changes increase surface roughness, promoting mechanical interlocking and stronger interfacial bonding between the fiber and the cement matrix^28^. This research employed a novel method by substituting 2.5%, 5%, 7.5%, and 10% of BSA with cement in the development of a cement-mortar mixture, thereby introducing a new approach for determining the optimal dosage of bamboo fiber. Furthermore, to explore the potential impact on the strength development, bamboo fiber was introduced into the binder within a spectrum varying from 1 to 4% in terms of weight. Recently separate research was executed on prefabricated bamboo reinforced panel with use of conventional mortar^29^. The novelty of this paper is to optimize the bamboo fiber reinforced mortar by using bamboo stem ash as a complementary binder and to incorporate the optimized bamboo fiber dosage in the prefabricated bamboo reinforced panel as a sustainable application to the wall panels.

## Materials

The materials used for this experimental research apart from conventional materials are bamboo fiber, bamboo stem ash and styrene butadiene rubber. Bamboo fibers extracted from leftover and waste bamboo pieces can affect the strength and durability of the fibers which leads to reduced strength, increased variability and decreased durability. To overcome these issues, mitigation strategies can be adopted which includes manual extraction of bamboo fiber that reduces damage and protect fiber integrity. Also, chemical treatment of fiber enhances its properties and durability. The bamboo fibers were extracted from leftover and waste bamboo pieces from the Chennai (Ambattur) local market, and the remaining stem pieces were incinerated to produce bamboo stem ash. Since the material used in this study was waste and discarded bamboo, no specific permissions or licenses were required for its collection. However, we confirm that the study did not involve harvesting live bamboo specimens. Bamboo fibers are extracted mechanically and treated with alkali solution before used as an additive in the mortar mix. Bamboo stem ash is used to replace cement material and the styrene butadiene rubber is used to enhance the property of mortar. The detailed description and properties of the research materials are as follows:

### Bamboo fiber

**Fig. 1:**
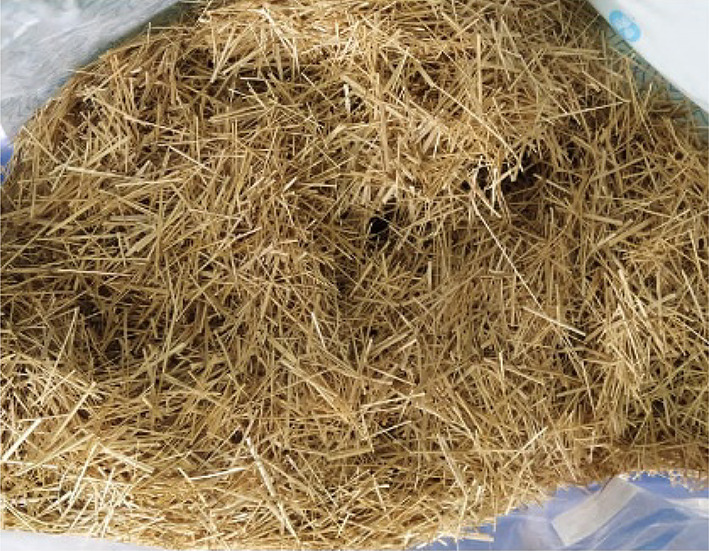
Manually extracted BF.

Based on the literature, bamboo strips were produced by mechanically extracting bamboo fibers from bamboo culms. To produce bamboo fibers, the strips were chopped based on the aspect ratio. The fiber had a diameter of 1 mm and length of 25 mm^30^. The cellulose content of the bamboo fiber was 73% and contained 13% hemicellulose. It also contained 12% lignin and 2% wax^31^ and the manually extracted BF is shown in Fig. 1.

**Fig. 2:**
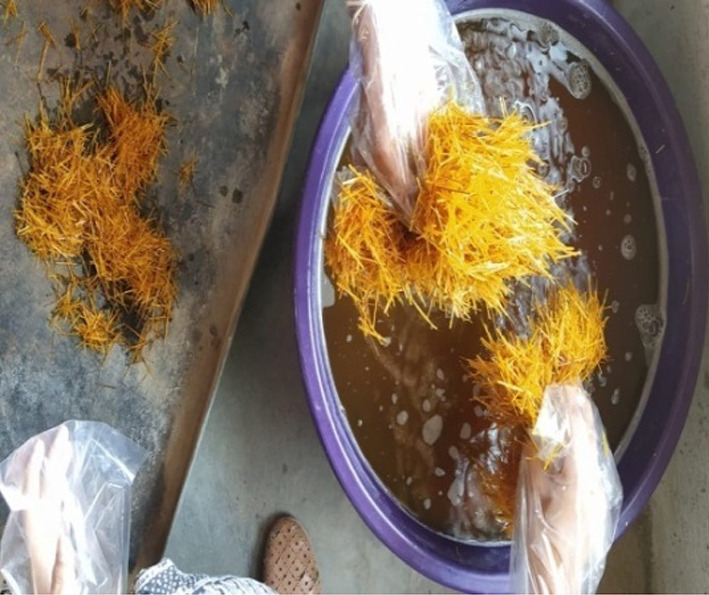
Removal of BF from NaOH solution.

**Fig. 3:**
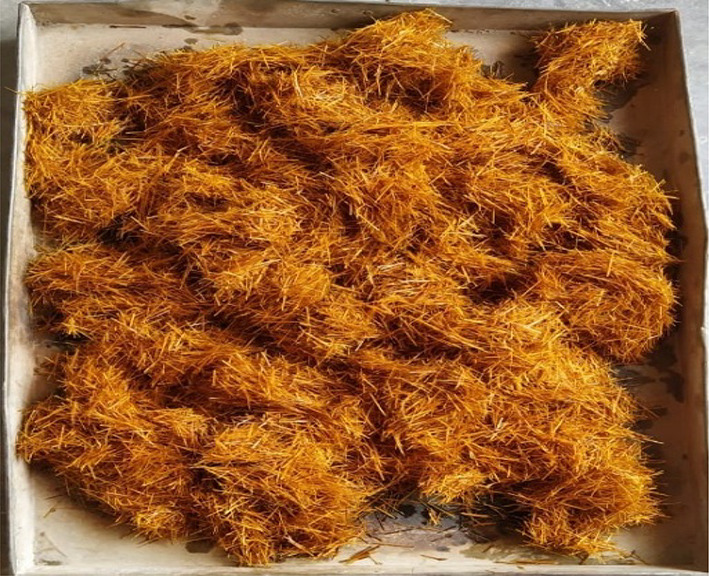
Treated bamboo fiber.

The bamboo fibers were removed, rinsed with tap water, and allowed to dry for 48 h. Subsequently, a laboratory grade sodium hydroxide (NaOH) solution (99.9% purity) was applied to the fiber to prevent voids from expanding owing to moisture content, which would have otherwise caused poor fiber matrix adherence. The surface roughness of natural fibers is increased by alkali treatment, which enhances their bonding and adhesion with the cement matrix. This treatment also reduces deterioration and improves durability by removing surface contaminants and waxes from the fibers. By eliminating weak points and flaws and aiding in the disintegration of fiber bundles, alkali treatment can increase the tensile strength of natural fibers while also enhancing their dispersion and distribution within the mortar matrix. Treated fibers can enhance mortar and concrete’s long-term performance and lower the chance of fiber deterioration. According to a previous study^32^, BF was immersed in a 10% NaOH solution for 48 h to improve interfacial bonding with the cement matrix. This alkaline treatment removes surface impurities such as waxes and hemicellulose, thereby increasing surface roughness and enhancing adhesion. After treatment, the fibers were thoroughly rinsed with tap water and sun-dried for two days, as shown in Figs. 2 and 3.

**Fig. 4:**
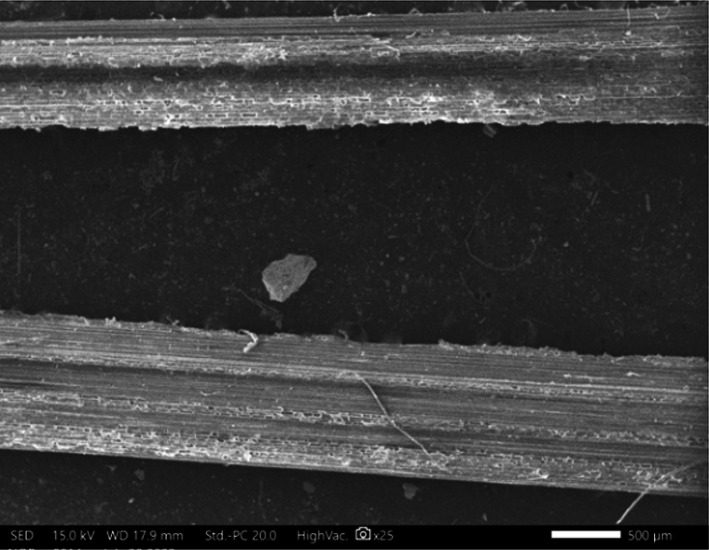
Morphology of treated BF.

Figure 4 presents the SEM (scanning electron microscope) morphology of NaOH-treated bamboo fiber, highlighting surface morphological changes due to alkali treatment. Although no direct measurements of fiber diameter were conducted in this study, existing literature^33,34^ indicates that alkali treatment with NaOH solution leads to the removal of hemicellulose and partial degradation of lignin, resulting in fibrillation and an apparent reduction in fiber diameter. The surface of the treated fiber also appears rougher due to the elimination of surface impurities and waxes. This increased surface roughness is considered beneficial for improving interfacial bonding in composite materials, as it enhances mechanical interlocking between the fiber and the binder matrix.

### Bamboo stem ash

**Fig. 5:**
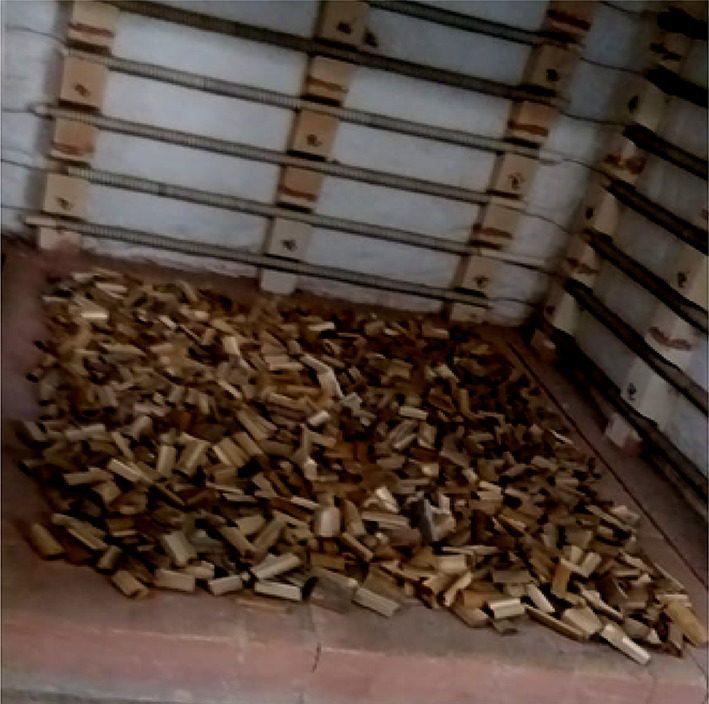
Chopped bamboo stem placed in furnace.

**Fig. 6:**
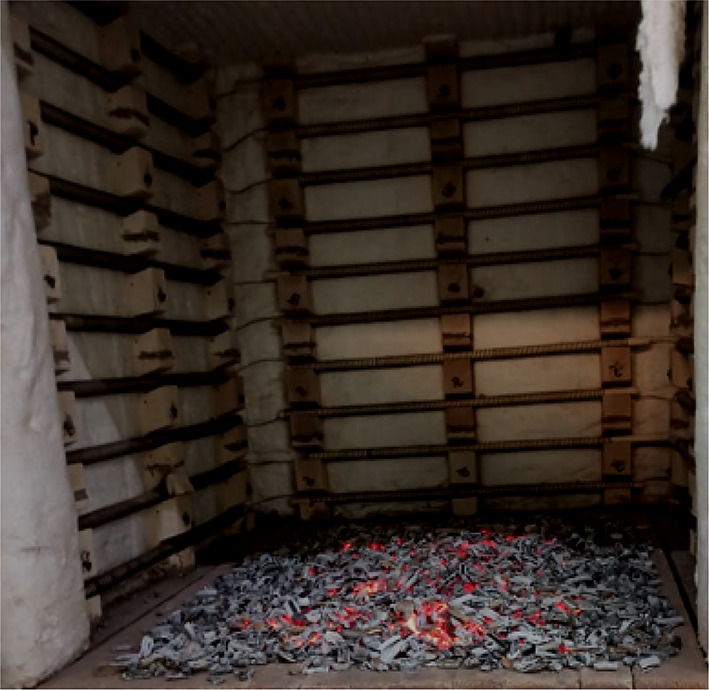
Incinerated bamboo stem in furnace.

The stem of the bamboo was divided into small pieces and allowed to dry entirely in the sun for 24 h to replace the cement with BSA. It was then placed in the furnace, as shown in Fig. 5, and burned for approximately 3 h at 600 °C^18^, as shown in Fig. 6. Using a 0.075 mm diameter sieve, the burned BSA was sieved to replace the OPC^18^. The chemical components of bamboo stem ash are listed in Table 1.

**Table 1:** Oxide composition of BSA from XRF analysis.

Chemical components (%)	BSA
SiO_2_	68.75
CaO	11.92
MgO	5.83
Al_2_O_3_	0.15
Fe_2_O_3_	0.16
Na_2_O	0.74
LOI	0.42

### Styrene butadiene rubber

Styrene butadiene rubber (SBR), a copolymer, was selected owing to its superior bonding properties. The specific gravity of the SBR is 1.04^35^. Furthermore, it offers excellent aging properties, crack resistance, and abrasion resistance. According to previous studies, mortar specimens containing 1.5% SBR had a greater compressive strength. The literature was used to calculate the range of SBR fixation (0.5–2%)^36^.

## Methodology

### Design of experiments using Taguchi approach

After determining the parameters and their levels, as discussed in the materials section, the experimental plan was developed using Taguchi’s design of experiments approach. Developed in the 1950s by Genichi Taguchi^37^, this statistical technique aims to improve product quality and process performance by systematically evaluating the effects of multiple variables with a reduced number of trials^38^. It enables efficient identification of key factors and their interactions using orthogonal arrays^39–41^. According to the Taguchi method, the total number of trial designs is expressed as N = LP, where P represents the parameters under consideration and L represents the levels for each parameter. An orthogonal array (OA) was used in the experimental design to limit the number of trials that could be run concurrently^42,43^. For the analysis of the experiment to be undertaken, the OA produced using this process are L4, L8, L9, L16, L18, L27, L32, etc. The OA of L16 was considered based on the selection of parameters and their values for this experimental work.

**Fig. 7:**
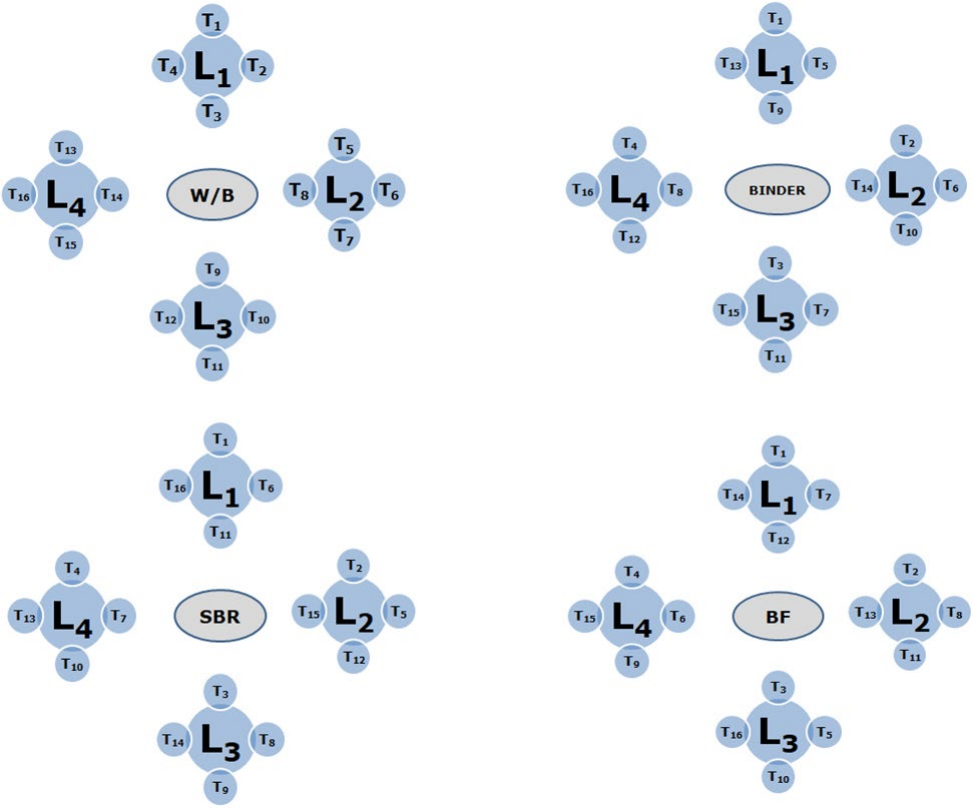
The arrangement of OA for all sixteen trials.

In the Taguchi technique, the degree of freedom methodology is used to analyze the least number of experiments that must be performed. There were four parameters, and four levels. Therefore, the maximum number of combinations is 44 or 256, but the orthogonal array L16 (number of levels × (number of parameters − 1) = 16) makes it simple to find the optimal value. All studies must be completed after selecting the OA and completing the trials in line with the level combinations. The dummy variable and interaction columns are crucial for comprehending the influence of interaction during data analysis, even though they are not necessary for carrying out the experiments. The parameters and levels for the experimental work are shown in Table 2. Figure 7 shows the arrangement of OA for all 16 trials created using this strategy and Table 3 shows the mix proportions of the mortar specimens as per Taguchi optimization. The quantity of materials as per mix proportions are listed for kg/m^3^ in Table 4.

**Table 2:** Variation levels and parameters.

Levels	W/B ratio	Binder (OPC + BSA) (%)	SBR (%)	BF (%)
L_1_	0.5	97.5 + 2.5	0.5	1
L_2_	0.45	95 + 5	1	2
L_3_	0.4	92.5 + 7.5	1.5	3
L_4_	0.35	90 + 10	2	4

**Table 3:** Mix proportion of control and trial specimens.

Exp. No	W/B	Binder (OPC + BSA) (%)	SBR (%)	BF (%)
Ctrl	0.5	100 + 0	0	0
T_1_	0.5	97.5 + 2.5	0.5	1
T_2_	0.5	95 + 5	1	2
T_3_	0.5	92.5 + 7.5	1.5	3
T_4_	0.5	90 + 10	2	4
T_5_	0.45	97.5 + 2.5	1	3
T_6_	0.45	95 + 5	0.5	4
T_7_	0.45	92.5 + 7.5	2	1
T_8_	0.45	90 + 10	1.5	2
T_9_	0.4	97.5 + 2.5	1.5	4
T_10_	0.4	95 + 5	2	3
T_11_	0.4	92.5 + 7.5	0.5	2
T_12_	0.4	90 + 10	1	1
T_13_	0.35	97.5 + 2.5	2	2
T_14_	0.35	95 + 5	1.5	1
T_15_	0.35	92.5 + 7.5	1	4
T_16_	0.35	90 + 10	0.5	3

**Table 4:** Quantity of materials as per mix proportions.

Exp. No	Cement (kg)	BSA (kg)	Water (litre)	SBR (litre)	BF (kg)
Ctrl	0.4992	0	0.2496	0	0
T_1_	0.486	0.00564	0.2458	0.00245	0.004916
T_2_	0.4742	0.01128	0.2427	0.00485	0.009708
T_3_	0.4617	0.01691	0.2393	0.00717	0.014358
T_4_	0.4492	0.02253	0.2358	0.00943	0.018868
T_5_	0.4862	0.00564	0.2212	0.00491	0.014742
T_6_	0.4742	0.01128	0.2184	0.00242	0.019416
T_7_	0.4617	0.01696	0.2153	0.09572	0.004786
T_8_	0.4492	0.02253	0.2122	0.00707	0.009434
T_9_	0.4865	0.00564	0.1966	0.00737	0.019664
T_10_	0.4742	0.01128	0.1941	0.00970	0.014562
T_11_	0.4617	0.01695	0.1914	0.00717	0.009572
T_12_	0.4492	0.02256	0.1886	0.00471	0.004717
T_13_	0.4865	0.00564	0.1720	0.00983	0.009832
T_14_	0.4742	0.01128	0.1698	0.00728	0.004854
T_15_	0.4617	0.01695	0.1675	0.00478	0.019144
T_16_	0.4492	0.02255	0.1650	0.00707	0.014151

The bamboo fiber-reinforced mortar was fabricated in accordance with ASTM C109 guidelines, employing a binder-to-fine aggregate (FA) ratio of 1:2.75 for various W/B ratios of, 0.50, 0.45, 0.40, and 0.30. Sixteen design mixes were formulated using Taguchi’s OA array, where BF and BSA were substituted for 1%, 2%, 3%, and 4% of the weight of the binder and 2.5%, 5%, 7.5%, and 10% of the cement weight, respectively. Table 3 lists the procedure used to prepare the sixteen experimental trial specimens. After identifying the optimum dosage with respect to dry density, water absorption, compressive and flexural strength, an experimental investigation was conducted to prove that optimum dosage of the Taguchi approach successfully satisfies ideal conditions.

### Sample Preparation

**Fig. 8:**
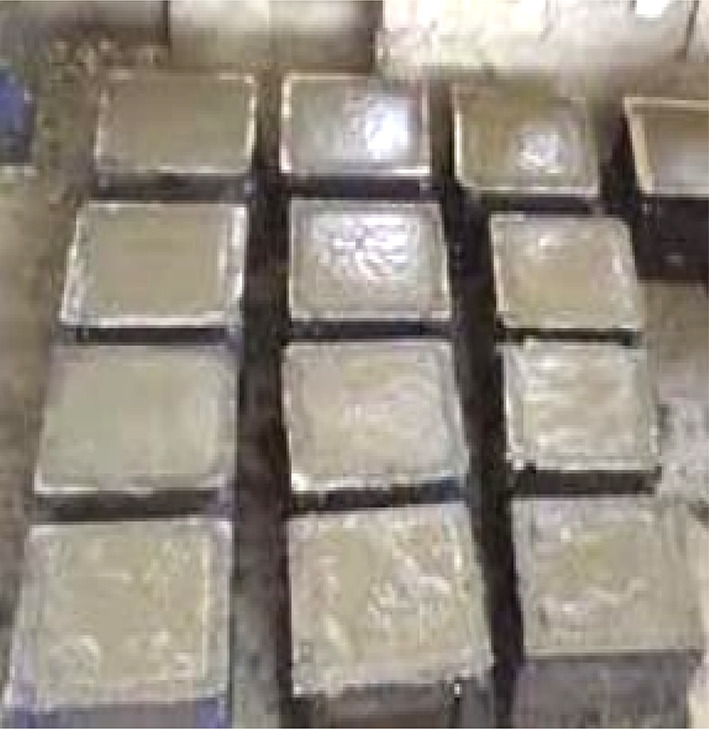
Casted cube specimen.

**Fig. 9:**
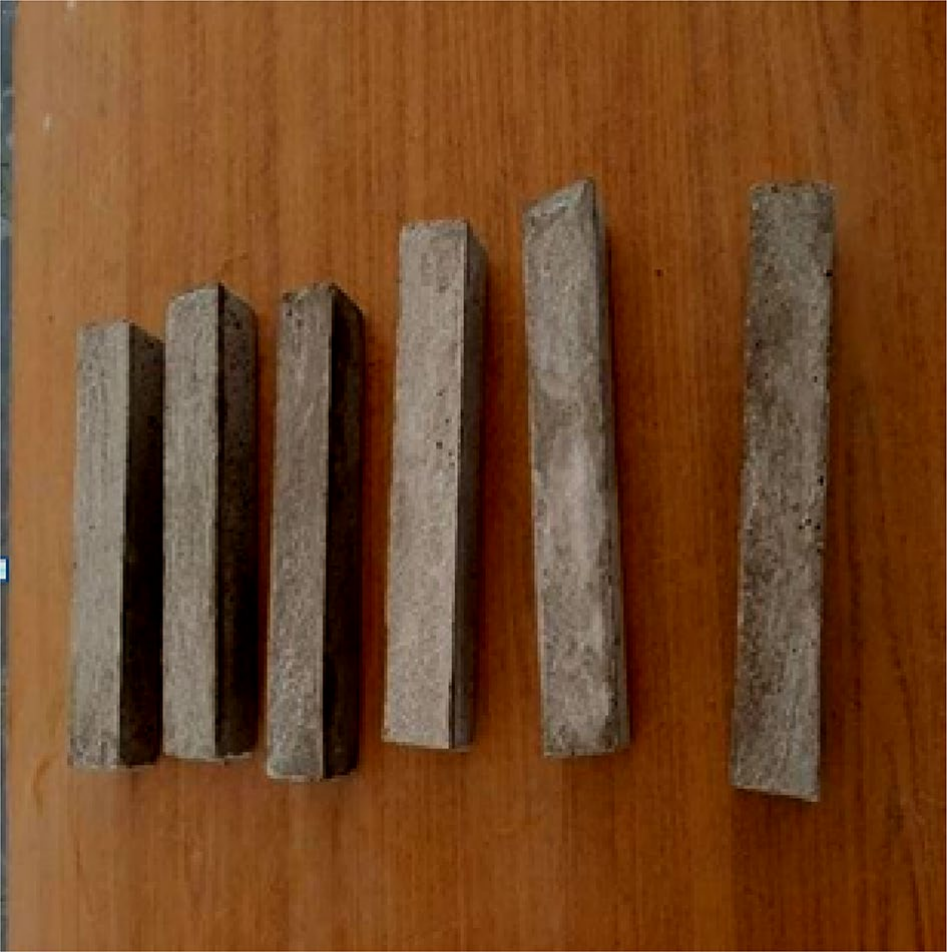
Casted prism specimen.

Following dry mixing, all components were weighed according to the mix proportions. Subsequently, water was gradually added while performing a wet mixing process for 2–3 min. The entire combination was placed in a flat container and manually stirred to prevent the fiber from separating in the mortar mix. Before pouring the fresh mortar mixture into molds sized at 100 × 100 × 100 mm³ and 40 × 40 × 160 mm³, the bamboo fibers were uniformly dispersed. Subsequently, the mortar was cured and hardened before testing for the compressive and flexural strengths. The cube and prism specimens obtained, after casting are shown in Figs. 8 and 9, respectively.

### Testing methods

The compressive strength of the produced cube specimens (100 × 100 × 100 mm^3^) was assessed after curing for seven and twenty-eight days using an ASTM C109-compliant compression testing apparatus with a 2000 kN capacity and an average of three samples for each trial. The load was gradually applied at a 0.75 kN/s loading rate. Prism samples (40 × 40 × 160 mm^3^) were subjected to flexural testing in accordance with ASTM C348 using universal testing machine (UTM) of 10 kN capacity under three-point loading condition. All sample cubes were physically tested for water absorption in accordance with the BS 1881 − 122 standard^44^. After 28 days, the mortar samples cured in water were retrieved and dried for 24 h at 100 °C in an oven. Subsequently, the oven-dried cube samples were weighed to determine their dry density. The test cube specimens were submerged in water for up to 240 min to assess the physical testing of water absorption. After removing the wet mortar samples from the water, they were weighed for mass at intervals of 10, 20, 30, 60, 120, 180, and 240 min^45^. The water absorption percentage was then computed.

## Results and discussions

### Computation of dry density with experimental results and Taguchi analysis

**Fig. 10:**
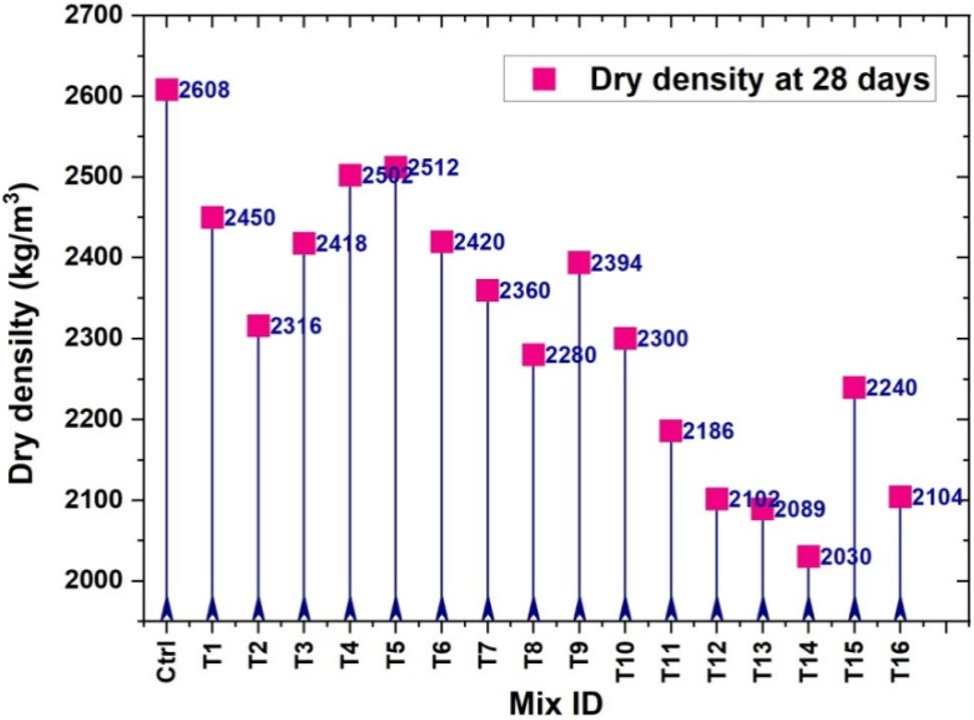
Dry density results of mortar specimens after 28 days of curing regime.

Figure 10 presents the experimental results for all control and trial mixes with different proportions of BF and BSA. The bulk density of BSA (651 kg/m³) is notably lower than that of ordinary Portland cement (1440 kg/m³)^18^. As the percentage of BSA in the mortar increases, a general reduction in dry density is observed. This reduction is primarily due to the lower density of BSA compared to conventional materials such as cement and sand. Replacing denser materials with lightweight bamboo ash leads to an overall lighter mix. Additionally, the porous structure of BSA may reduce packing density, further lowering the mortar’s bulk density. Although a lower density can provide benefits such as improved thermal insulation, it is essential to ensure that strength and durability are not compromised for the intended use. Interestingly, an increase in dry density was noticed in mixes containing 7.5–10% BSA, which can be attributed to the presence of 3–4% BF. The observed dry density values varied between 2030 and 2512 kg/m³. The mix containing 5% BSA and 1.5% BF recorded the lowest dry density, with a 22.8% reduction compared to the control specimen. The dry density remained unchanged when the styrene-butadiene rubber (SBR) content was increased to 1%; however, a gradual rise in dry density was noticed when SBR content exceeded 1.5%.

**Fig. 11:**
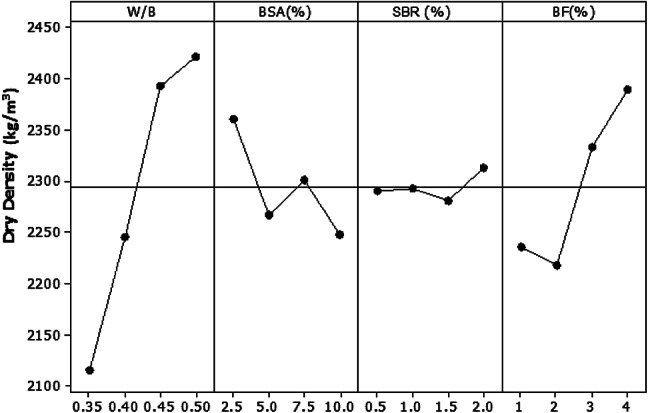
Main dry density effect plot at 28 days.

Figure 11 illustrates the main effect plot for dry density based on the Taguchi analysis. In this study, the goal was to achieve a lower dry density to promote lightweight cement mortar suitable for sustainable structural applications. Therefore, the appropriate optimization criterion used was “the smaller is better”^38^. According to the analysis, the water-to-cement ratio exhibited the most significant influence on dry density, contributing 66.73% to the overall variation. The incorporation of BF showed the next highest contribution at 21.98%, followed by BSA and SBR. The findings reveal that a lower w/c ratio leads to a reduced dry density, which is consistent with the intention to develop lightweight materials. The optimal combination for achieving the desired reduction in dry density was identified as follows: a water-to-cement ratio of 0.35, 10% BSA, 1.5% SBR, and 2% BF.

### Water absorption characteristics and main effect plot by Taguchi analysis

**Fig. 12:**
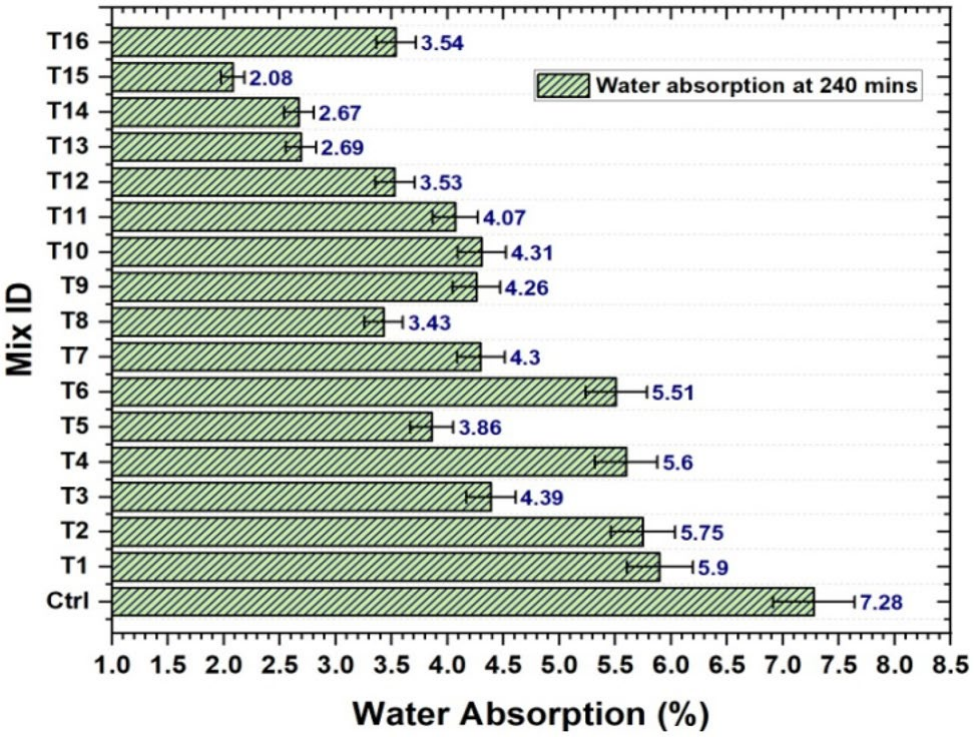
Water absorption results of mortar specimens at 28 days.

The water molecules had a place to stay because of their porous structure. The amount of bamboo fiber present appears to increase in direct proportion to the increase in mortar moisture content. However, the water absorption percentage of the sample mortar decreased as a result of alkali treatment of the bamboo fiber with NaOH before being added to the mortar mixture^32^. The alkali treatment improves the surface properties of the bamboo fibers by eliminating lignin, hemicellulose, and other contaminants. By strengthening the link between the fibers and the mortar matrix, this change can create a structure that is denser and less porous. Fibers that have been treated often mix in the cement matrix better. This may lessen the quantity of spaces or air pockets in the mortar, which will lessen the mortar’s ability to absorb water. The fibers’ physical structure may change because of the alkali treatment, decreasing their porosity. As a result, the mortar’s overall water absorption is reduced. Alkali treatment may occasionally give the fibers hydrophobic qualities, which will reduce their absorption of water even further. The water absorption percentage of the control mortar sample was 7.28%, whereas, that of the trial mortar samples ranged from 2.08 to 5.89%. Figure 12 shows the water absorption percentage at 240 min for various mixes after 28 days. From this experimental result, it is observed that the minimum and maximum decreases in the level of absorption of water for the trial mortar samples are 5.21% and 1.4% respectively.

**Fig. 13:**
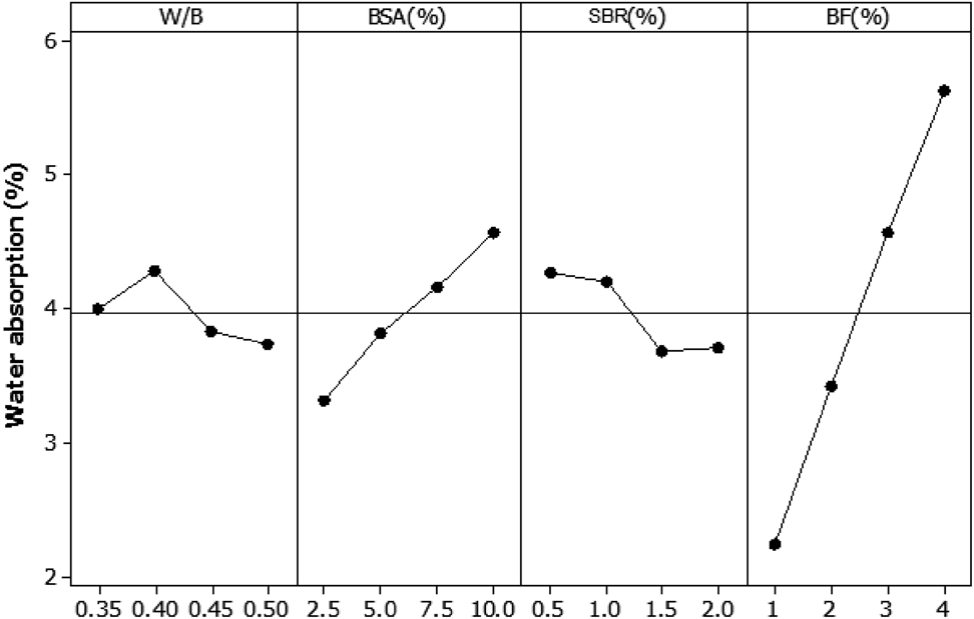
Main water absorption effect plot at 28 days.

For each of the sample mortars, the water absorption percentage decreased because of the NaOH treatment customized for the bamboo fiber prior to its addition to the mortar mixture. BF had the largest impact on water absorption, contributing 81.67% of the total. Figure 13 shows the plot of the main water absorption effect. The water absorption percentage gradually increased as the BF percentage increased. Additionally, the percentage of water absorption increased owing to the replacement of BSA. BSA contributed 10.71%, making it the second most important factor in water absorption property. Therefore, based on the analysis, 0.5 W/B, 1.5% SBR, 2.5% BSA and 1% BF are the ideal water absorption ratios.

### Progression of compressive strength and main effect plot by Taguchi analysis

**Fig. 14:**
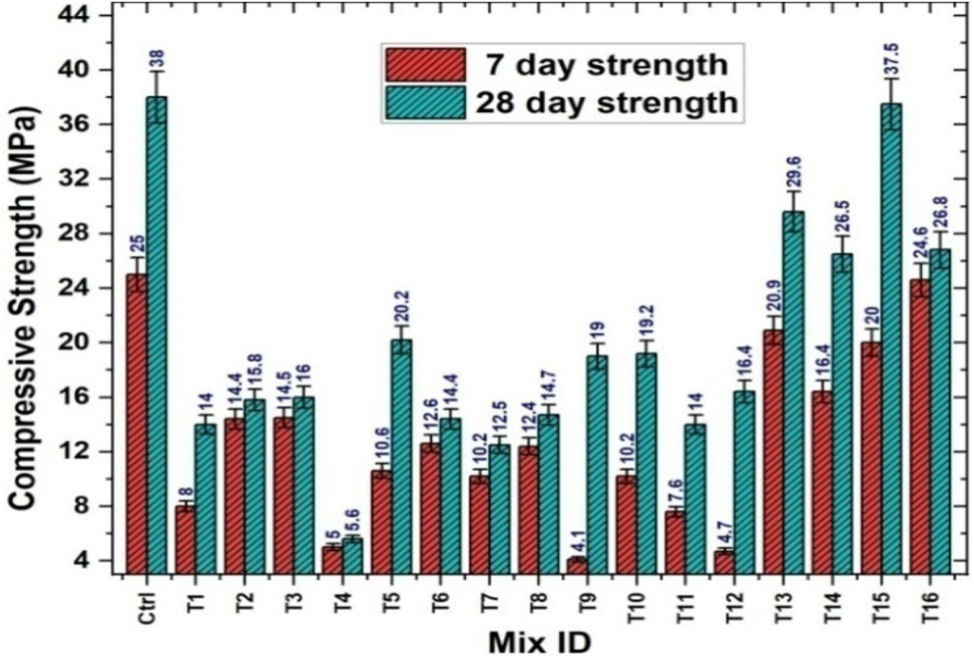
Compressive strength results of mortar specimens.

The experimental results clearly demonstrate that the water-to-binder ratio significantly influences the compressive strength of the mortar. As the W/B ratio decreased, a progressive increase in compressive strength was observed. This improvement can be attributed to reduced capillary porosity, resulting from limited excess water in the mixture. At lower W/B ratios, better particle packing and denser matrix formation are achieved, contributing to enhanced mechanical performance. Furthermore, reduced water content restricts the development of voids formed during evaporation, thereby decreasing the overall porosity. Although a certain amount of water is essential to facilitate the hydration process, excessive water tends to dilute the binder matrix, weakening the internal structure. A lower W/B ratio ensures more efficient use of the available water for hydration, leading to a stronger and more cohesive microstructure. As a result, the hardened mortar exhibits reduced permeability and improved durability, alongside increased compressive strength. In addition, the combined application of treated BF and SBR was observed to enhance the compressive strength of the mortar specimens. As illustrated in Fig. 14, compressive strength values for various mixes at 7 and 28 days ranged from a minimum of 5.6 MPa (Trial 4) to a maximum of 37.5 MPa (Trial 15), reflecting the influence of mix proportions on strength development.

**Fig. 15:**
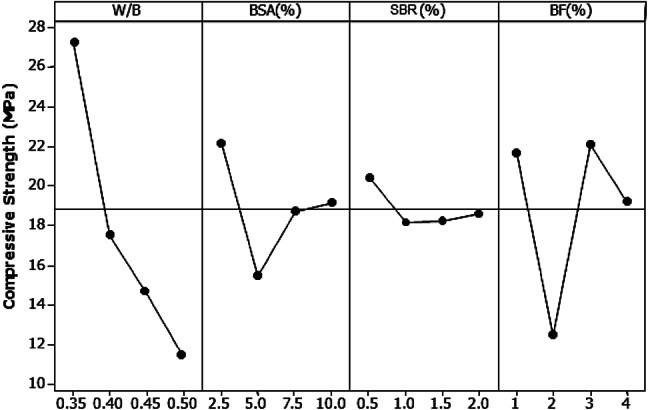
Main compressive strength effect plot at 28 days.

The significance of the W/B ratio in determining compressive strength is shown in Fig. 15. A gradual decrease in the W/B ratio was correlated with an increase in compressive strength. The W/B ratio accounted for 54.04% of the variation in compressive strength. Following this, BF contributes 25.80%, ranking second after the W/B ratio in terms of its influence on the compressive strength of the mortar. Based on the analysis, the optimal dosage for enhancing the compressive strength includes 2.5% BSA, a water-to-binder ratio of 0.35, 0.5% SBR, and 3% BF.

### Progression of flexural strength and main effect plot by Taguchi analysis

**Fig. 16:**
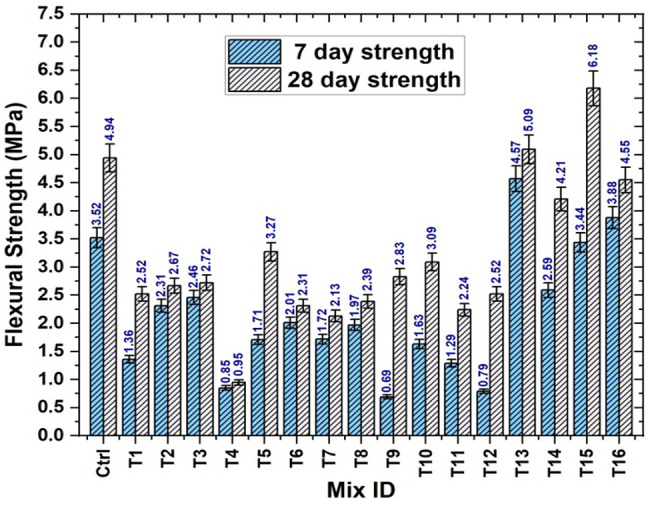
Flexural strength results of prism specimens.

Similar to the compressive strength, the flexural strength of the prism specimens was influenced by the W/B ratio. The water-to-binder ratio affected the flexural strength of the prism specimen in a manner similar to that of the compressive strength. The flexural strength of the prism specimens increased as the ratio decreased. A denser microstructure produced by a lower W/B ratio increases the material’s overall strength. This density lessens the chance of cracking and failure by helping to distribute loads during flexural loading more effectively. The binding between the aggregates and binder is stronger when there is less water in the mixture. Higher flexural strength is the result of improved load transfer across the material caused by stronger linkages. In general, a structure that is denser and less porous is more resilient to the spread of cracks. Because it aids in the material’s integrity and strength preservation under loading circumstances, this resistance is essential under flexural stress. The increased flexural strength of the specimen was largely attributable to the addition of treated bamboo fiber. The flexural strengths of the control and trial specimens at 7 and 28 days are shown in Fig. 16. From the experimental values obtained, the minimum and maximum strengths of the specimens were observed in trials 4 (0.95 MPa) and 15 (6.18 MPa), respectively.

**Fig. 17:**
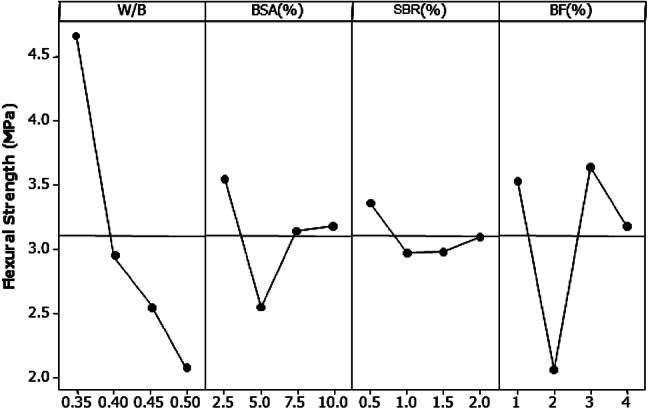
Main flexural strength effect plot at 28 days.

Figure 17 clearly indicates the significant impact of the W/B ratio on the flexural strength of the mortars. The W/B ratio contributed 56.03% of the total flexural strength. The flexural strength increased gradually with a decrease in the W/B ratio. Consequently, BF contributed 24.53% to the flexural strength of the mortar. According to the analysis of variance, the optimal combination for enhancing flexural strength includes a water-to-binder (W/B) ratio of 0.35, 2.5% bamboo stem ash (BSA), 0.5% superplasticizer, and 3% bamboo fiber (BF).

Table 5 presents the variance of the mortars derived from the Taguchi analysis, while Table 6 provides the tabulated ideal mix proportions extracted from Figs. 11, 13, 15 and 17. Despite the varying responses of the mortar under tension and compression loads, a consistent optimal proportion of bamboo fibers in the mortar were attained. Selecting ideal proportions of dry density and water absorption may be advantageous for structures designed with reduced weight and enhanced durability.

**Table 5:** Examination of mortar variance.

Indexes of performance	Parameter	Degree of freedom	Sum of squares	Mean square	Contribution (%)
Dry density	W/B	3	240,730	80243.4	66.73
	BSA (%)	3	30,147	10049.1	8.35
	SBR (%)	3	2208	736.1	0.61
	BF (%)	3	79,286	26428.7	21.98
	Residual	3	8341	2780.4	2.31
	Total	15	360,713	120237.7	100
Water absorption	W/B	3	0.6798	0.2266	2.15
	BSA (%)	3	3.3721	1.1240	10.71
	SBR (%)	3	1.1557	0.3852	3.67
	BF (%)	3	25.7121	8.5707	81.67
	Residual	3	0.5632	0.1877	1.78
	Total	15	31.4829	10.4942	100
Compressive strength	W/B	3	492.02	164.008	54.04
	BSA (%)	3	89.56	29.854	9.83
	SBR (%)	3	13.96	4.654	1.53
	BF (%)	3	234.90	78.301	25.80
	Residual	3	79.99	26.662	8.78
	Total	15	910.44	303.479	100
Flexural strength	W/B	3	14.2341	4.7447	56.03
	BSA (%)	3	2.0362	0.6787	8.01
	SBR (%)	3	0.3904	0.1301	1.53
	BF (%)	3	6.2324	2.0775	24.53
	Residual	3	2.5077	0.8359	9.87
	Total	15	25.4008	8.4669	100

**Table 6:** Ideal mortar mix proportions.

Combined indices	BSA (%)	SBR (%)	BF (%)	W/B ratio
Compressive strength	2.5	0.5	3	0.35
Flexural strength	2.5	0.5	3	0.35
Dry density	10	1.5	2	0.35
Water absorption	2.5	1.5	1	0.5

It was tested experimentally to determine whether the obtained ideal mix proportion indeed resulted in minimal values for water absorption and dry density, and maximal values for compression and flexural strength. This was done to show how Taguchi’s method yielded the best mix proportion dosage. The experimental findings shown in Table 7 prove that optimum dosage of the Taguchi approach successfully satisfies ideal conditions.

**Table 7:** Results from experiments on the ideal mix ratios.

Mortar property	Sample ID	Experimental results
Dry density (kg/m^3^)	OM-1	2042
Water absorption (%)	OM-2	1.52
Compressive strength (N/mm^2^)	OM-3	37.4
Flexural strength(N/mm^2^)	OM-3	6.13

### Micro structural characterization on optimal mix combination

**Fig. 18:**
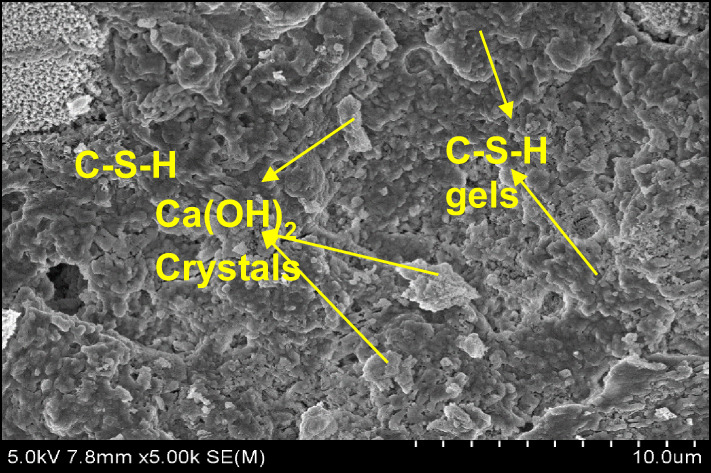
SEM image of mortar (OM-1).

**Fig. 19:**
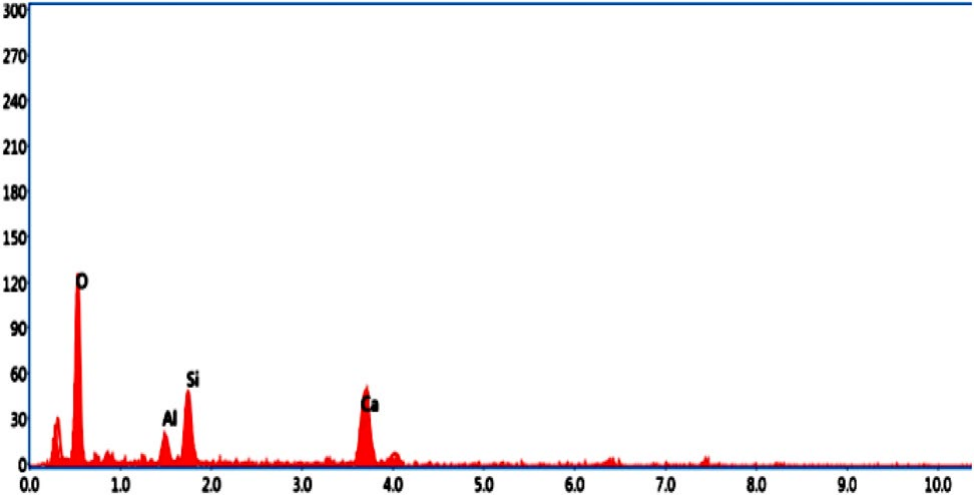
EDS element composition graph of OM-1.

**Fig. 20:**
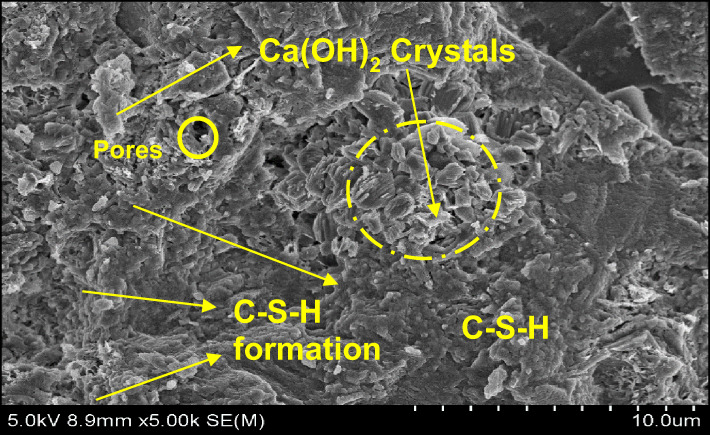
SEM image of mortar (OM-2).

**Fig. 21:**
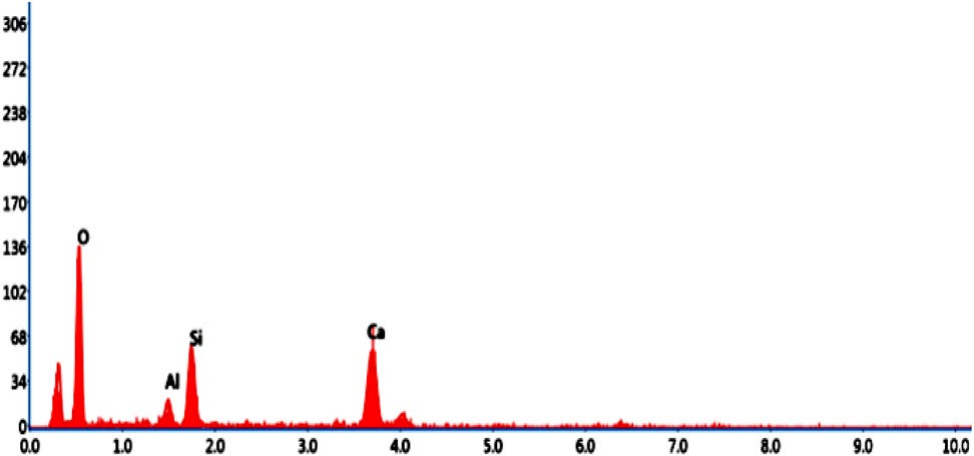
EDS element composition graph of OM-2.

**Fig. 22:**
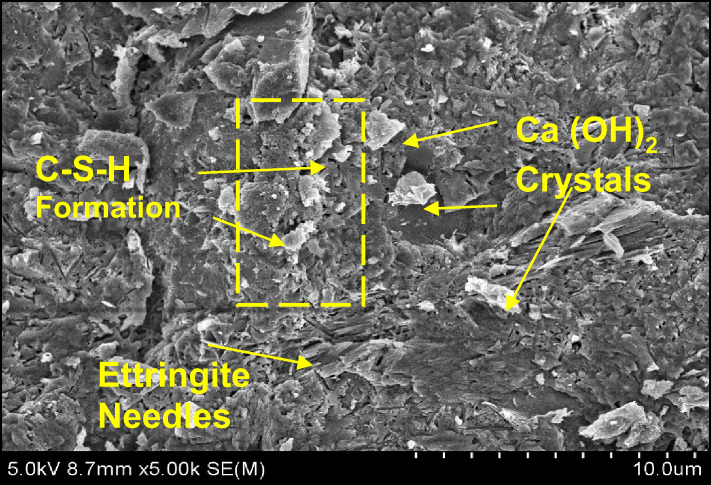
SEM image of mortar (OM-3).

**Fig. 23:**
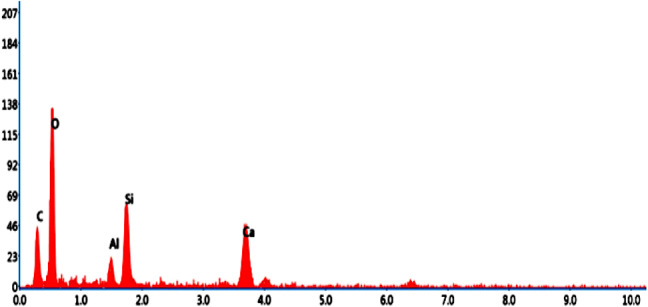
EDS element composition graph of OM-3.

The authentication of optimized reinforced concrete with bamboo fiber using bamboo stem ash as an alternative binder on the physical and mechanical performance was studied using SEM analysis and energy dispersive spectroscopy (EDS). The significant configurations of both hydrated and un-hydrated states of the optimized mortar with respect to water absorption, dry density, flexural strength, and compressive strength are shown in Figs. 18, 19, 20, 21, 22 and 23.

C-S-H appears as an amorphous, gel-like phase. Its structure could be layered or fibrous. It usually has a texture that is thick and slightly grainy. It may also show up as a web of minuscule particles. Its color appears somewhat transparent and is frequently paler than Ca(OH)₂. Compared to C-S-H, Ca(OH)₂ appears as more distinct, crystalline particles. It frequently has a more angular, needle- or plate-like shape. It can manifest as bigger, more solitary crystals or clusters. It is larger than C-S-H on average, having micrometer-scale dimensions. In SEM photos, it typically appears white or light gray. Similar to C-S-H, C-S-H gels may exhibit more complicated structures as a result of varied polymerization intensities or larger water contents. It frequently has a less thick and more porous appearance than crystalline Ca(OH)_2_. Like C-S-H, the size is in the nano-scale range, but because of its gel-like consistency, it might have more asymmetrical shapes^46–48^.

In comparison with all the optimized mortar specimens, the scanning electron microscope image of (OM-3) corresponding to flexural strength and compressive strength as shown in Fig. 22 exhibited a higher configuration of calcium silicate hydrate (C-S-H) gel, calcium hydroxide (Ca(OH)_2_) crystal formation, and ettringite needles with the cement matrix. In addition, the treated bamboo fiber concentration of 3%, improved the homogeneity of the mix and increased the bonding characteristics. Bamboo stem ash in place of ordinary Portland cement by 2.5% made the particles less reactive and densely packed, thereby contributing to the completion of the hydration process. These are the significant reasons why the OM-3 mix achieves higher compressive and flexural strengths than the other mortar mixes. The EDS elemental composition of OM-3 is shown in Fig. 23.

In Fig. 20, it is observed that the surface morphology is closely packed, and significant ettringite needles and accumulation of C-S-H gel are visible. Bamboo stem ash, in place of ordinary Portland cement by 2.5%, made the particles less reactive and densely packed, thereby contributing to the completion of the hydration process. In addition, the treated bamboo fiber with a concentration of 1% increased the bonding characteristics and maintained small voids and micro cracks. These are the authorized reasons for the resistance of OM-2 mix towards water absorption. The EDS elemental composition of OM-2 is shown in Fig. 21.

A similar surface morphology was observed in OM-1 (Fig. 18), pertaining to the dry density. Bamboo stem ash in place of OPC decreased the cement content by 10%, which led to a lower hydration process. A treated bamboo fiber concentration of 2% improved the homogeneity of the mix and increased the bonding characteristics, thereby reducing the density of the mortar. The EDS elemental composition of OM-1 is shown in Fig. 19.

**Table 8:** Element distribution from EDS investigation.

Components	OM-1	OM-2	OM-3
O	52.42	50.93	47.44
Ca	32.61	33.72	24.95
Si	10.62	11.48	11.5
Al	4.35	3.87	3.56
C	–	–	12.56
Ca/Si	3.07	2.93	2.16

The elemental composition of the optimized mortar mixes from the EDS assessment at a particular point is shown in Table 8. The EDS assessment of the optimized mortar specimens predominantly contained oxygen and calcium, and as well as some constituents of silica and alumina, which is the main reason for the generation of Ca(OH)_2_ and C-S-H gels from during the hydration process. In addition, the intensity of the hydration process was determined by the ratio of calcium to silica (Ca/Si). When Ca/Si proportion lies between 3 and 4, it means the commencement of the reaction of hydration. A Ca/Si ratio of less than 3, signifies completion of the hydration reaction^49^.

Under conditions of accelerated weathering, brickwork reinforced with bamboo fibers may eventually deteriorate significantly. The performance of the bamboo fiber reinforced mortar is influenced by carbonation, microbial attack, and UV exposure. Bamboo fibers may deteriorate under UV light, losing some of their mechanical qualities. According to studies, bamboo fiber-reinforced composites’ flexural strength can be lowered by up to 7% when exposed to UV light. Micro cracks, voids, and surface abrasion can also result from UV penetration and ongoing moisture absorption. Bamboo fibers may degenerate due to microbial development, jeopardizing the integrity of the composite. Alkaline Copper Quaternary (ACQ) is one therapy that can offer defense against microbial deterioration. By changing the pH levels and possibly causing fiber breakdown, carbonation can shorten the lifespan of mortar reinforced with bamboo fibers. To completely comprehend the long-term aging effects on these composites, more investigation is required.

### Energy efficiency

**Fig. 24:**
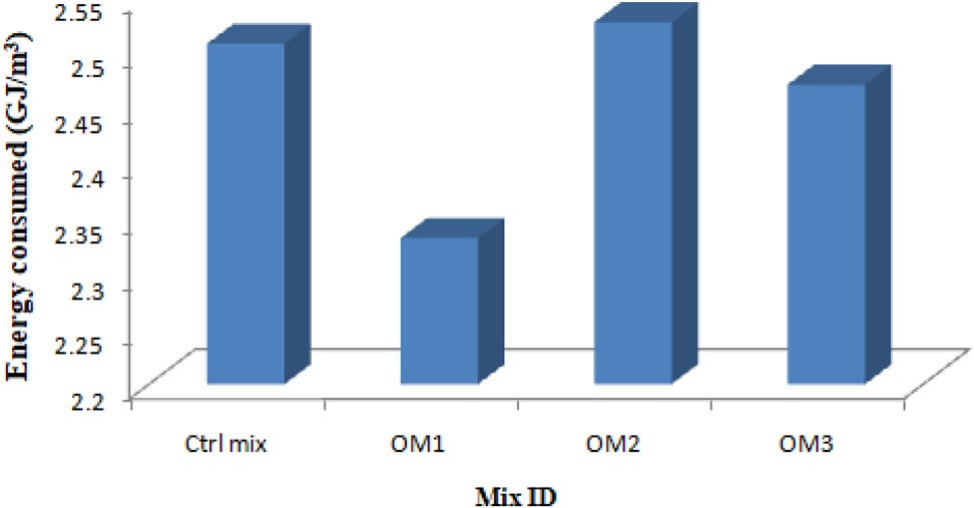
Energy efficiency.

Energy efficiency is the main factor that influences sustainability. Energy efficiency is determined by comparing the strength demonstrated to the total energy used to produce the various constituents, including the matrix’s copper slag, cement, BSA, river sand, and SP. The energy efficiency is obtained from the ratio of strength and energy consumed to produce one cubic meter. Figure 24 indicates the energy efficiency of optimum mortar specimens.

**Fig. 25:**
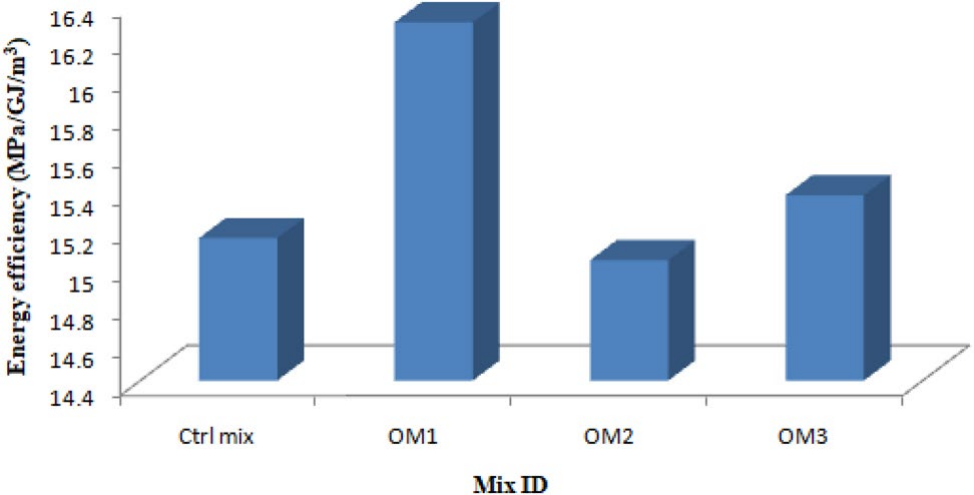
Energy consumption.

One ton of OPC and one ton of BSA require 4.8GJ^50^ and 0.034GJ^51^ of energy, respectively. The amount of energy required for the production of one ton of fine aggregate is 0.081GJ^52^. Copper slag is a waste material produced by refinery plants during the extraction process of copper metal and hence it does not consume any extra energy for the production^50^. The energy consumption for the production of one ton of SBR and water is 11.5GJ and 0.2GJ respectively^50^. The energy consumption of BF is not included since it was extracted manually. The quantity of materials and energy consumed of all the materials of optimum mixes is shown in Table 9. The energy efficiency (EE) of the control and optimum mixes is shown in Table 10. The energy consumed and the energy efficiency graph is shown in Figs. 25 and 24 respectively.

**Table 9:** Energy consumption of mixes.

Ingredients	Quantity (kg)	Energy consumed (GJ/m^3^)
Ctrl Mix		
OPC	499.201	2.396
BSA	0.000	0.000
Water	249.612	0.04992
SBR	0.000	0.000
Sand	762.010	0.061722
Copper Slag	1101.025	–
BF	0.000	–
Total		2.507642
OM-1		
OPC	449.212	2.156
BSA	22.521	0.000765
Water	165.000	0.033
SBR	7.010	0.0805
Sand	762.010	0.061722
Copper Slag	1101.025	–
BF	9.411	–
Total		2.331987
OM-2		
OPC	486.0	2.332
BSA	5.6	0.0001904
Water	245.8	0.04916
SBR	7.3	0.08395
Sand	762.010	0.061722
Copper Slag	1101.025	–
BF	4.921	–
Total		2.5270224
OM-3		
OPC	486.0	2.332
BSA	5.6	0.0001904
Water	245.8	0.04916
SBR	2.4	0.0276
Sand	762.010	0.061722
Copper Slag	1101.025	–
BF	14.732	–
Total		2.4706724

**Table 10:** Energy efficiency of mixes.

Sample ID	Energy consumed (GJ/m^3^)	Energy efficiency (MPa/ GJ/m^3^)
Ctrl mix	2.507642	15.1536
OM-1	2.331987	16.2951
OM-2	2.5270224	15.0374
OM-3	2.4706724	15.3808

### CO_2_ efficiency

**Fig. 26:**
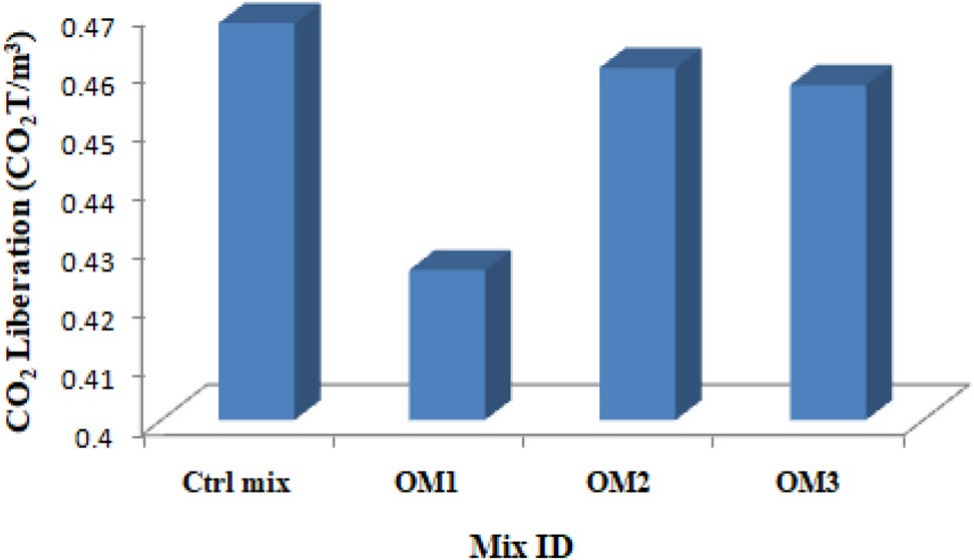
CO_2_ Liberation.

The second most significant variable that affects sustainability is CO_2_ efficiency. Fuel combustion, which provides the energy needed for the synthesis of various materials, releases carbon dioxide. Fine aggregate production releases the least amount of CO_2_, at 0.0048 tonnes per ton of production. OPC emits 0.93 ton of CO_2_ for every one ton of production. The amount of CO_2_ released by SBR and water is 0.6 ton and 0.0008 tonne respectively for every one ton of production. It was believed that the CO_2_ output from burning the bamboo stem to generate BSA could be absorbed during the life cycle of bamboo plants, hence it was not taken into account^53^. Figure 26 shows the CO_2_ emission calculation for each cubic meter of control and optimum mixes.

**Fig. 27:**
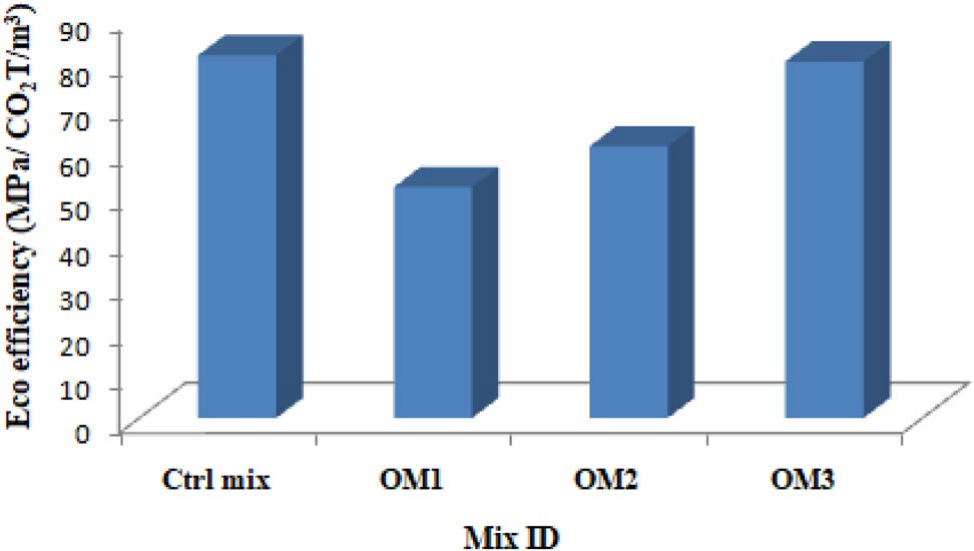
Eco efficiency.

It is clear from Fig. 26 that using BSA results in less CO_2_ being released into the atmosphere. Figure 27 illustrates how eco efficiency is computed, which is equivalent to energy efficiency, using the strength to CO_2_ emission ratio. The eco efficiency is obtained from the ratio strength and carbon dioxide liberated to produce one cubic meter. The quantity of materials and CO_2_ emission of all the materials of optimum mixes is shown in Table 11. The eco efficiency of the control and optimum mixes is shown in Table 12.

**Table 11:** CO_2_ liberation of mixes.

Ingredients	Quantity (kg)	CO_2_ Liberation (CO_2_T/m^3^)
Ctrl Mix		
OPC	499.201	0.464
BSA	0.000	–
Water	249.612	0.00019968
SBR	0.000	–
Sand	762.010	0.0036576
Copper Slag	1101.025	–
BF	0.000	–
Total		0.46785
OM1		
OPC	449.212	0.41775
BSA	22.521	–
Water	165.000	0.000018
SBR	7.010	0.0042
Sand	762.010	0.0036576
Copper Slag	1101.025	–
BF	9.411	–
Total		0.42562
OM2		
OPC	486.0	0.45198
BSA	5.6	–
Water	245.8	0.00019664
SBR	7.3	0.00438
Sand	762.010	0.0036576
Copper Slag	1101.025	–
BF	4.921	–
Total		0.46021
OM3		
OPC	486.0	0.45198
BSA	5.6	–
Water	245.8	0.00019664
SBR	2.4	0.00144
Sand	762.010	0.0036576
Copper Slag	1101.025	–
BF	14.732	–
Total		0.45727

**Table 12:** Eco efficiency of mixes.

Sample ID	CO_2_ Liberation (CO_2_T/m^3^)	Eco efficiency (MPa/ CO_2_T/m^3^)
Ctrl mix	0.46785	81.22
OM1	0.42562	51.68
OM2	0.46021	60.84
OM3	0.45727	79.83

Several challenges prevent bamboo stem ash-based mortar from being widely used in the construction industry. The ideal replacement percentage, variations in bamboo ash characteristics, long-term performance and durability, standardization and criteria, a lack of research and development, and awareness and acceptance are some of the major obstacles. Notwithstanding these difficulties, mortar made from bamboo stem ash has demonstrated encouraging outcomes in terms of increased durability, decreased density, and environmental advantages.

## Conclusions

This study investigated the incorporation of waste bamboo materials such as BF and BSA into cement mortar to develop a sustainable, high-performance alternative to conventional construction materials. The findings revealed that treatment of BF with 10% NaOH significantly reduced water absorption, improving the overall durability of the mortar, with values decreasing from 12.19% in the control mix to as low as 1.52% in the optimized mix. The inclusion of BSA, due to its lower density relative to cement, contributed to a reduction in dry density. The optimized mix, comprising 10% BSA, 1% BF, 1.5% SBR, and a water-to-binder ratio of 0.35, exhibited a 21.7% decrease in density, making it suitable for lightweight structural applications. Despite general strength reductions at higher BF and BSA contents, the optimized formulation with 2.5% BSA, 3% BF, and 0.5% SBR at a 0.35 W/B ratio resulted in a substantial increase in compressive and flexural strengths by 98.42% and 24%, respectively, compared to the control mix. The Taguchi method proved effective in optimizing performance parameters with fewer experimental trials, although advanced tools such as response surface methodology or machine learning could further enhance predictive capabilities.

## Potential applications and future scope

From a sustainability perspective, partial cement replacement with BSA supports circular economy goals by diverting waste, conserving raw materials, and reducing environmental impact. The developed BF–BSA based mortar is highly suited for prefabricated wall panels and modular construction, though further studies involving long-term durability, thermal analysis, and life cycle assessment are recommended to validate its full-scale applicability.

## Data Availability

The datasets analyzed during the current study are available from the corresponding author on reasonable request.
